# Willingness to participate in in-the-moment surveys triggered by online behaviors

**DOI:** 10.3758/s13428-022-01872-x

**Published:** 2022-05-31

**Authors:** Carlos Ochoa, Melanie Revilla

**Affiliations:** Research and Expertise Centre for Survey Methodology, Universitat Pompeu Fabra, Barcelona, Spain

**Keywords:** In-the-moment surveys, Meter, Online behavioral data, Passive data collection, Web surveys, Willingness to participate

## Abstract

Surveys are a fundamental tool of empirical research, but they suffer from errors: in particular, respondents can have difficulties recalling information of interest to researchers. Recent technological developments offer new opportunities to collect data passively (i.e., without participant’s intervention), avoiding recall errors. One of these opportunities is registering online behaviors (e.g., visited URLs) through tracking software (“meter”) voluntarily installed by a sample of individuals on their browsing devices. Nevertheless, metered data are also affected by errors and only cover part of the objective information, while subjective information is not directly observable. Asking participants about such missing information by means of web surveys conducted in the moment an event of interest is detected by the meter has the potential to fill the gap. However, this method requires participants to be willing to participate. This paper explores the willingness to participate in in-the-moment web surveys triggered by online activities recorded by a participant-installed meter. A conjoint experiment implemented in an opt-in metered panel in Spain reveals overall high levels of willingness to participate among panelists already sharing metered data, ranging from 69% to 95%. The main aspects affecting this willingness are related to the incentive levels offered. Limited differences across participants are observed, except for household size and education. Answers to open questions also confirm that the incentive is the key driver of the decision to participate, whereas other potential problematic aspects such as the limited time to participate, privacy concerns, and discomfort caused by being interrupted play a limited role.

## Introduction

Surveys are a major tool of empirical research (Saris and Gallhofer, 2014). However, they suffer from well-known errors (Groves et al., 2009) that can affect the results, sometimes leading to wrong conclusions (Saris and Revilla, 2016).

One major source of errors is related to the limitations of human memory (Tourangeau, 1999). Respondents may have difficulties self-reporting objective data related to events of interest to researchers (e.g., which websites did you visit yesterday?). Furthermore, subjective data are also affected by recall errors. Indeed, memories associated with negative emotions tend to be forgotten more quickly than those associated with positive ones (“Fading Affect Bias”; Walker and Skowronski, 2009). Moreover, the way people experienced events and the way they remember them may differ significantly (Kahneman, 2011).

Although surveys have significantly evolved over the past decades thanks to the use of new technologies, mainly to adapt to online data collection (Lumsden, 2007) and through mobile devices (Mavletova and Couper, 2015), recall errors are still a key issue in current (mobile web) surveys.

New technologies also offer alternatives that collect data without relying on people’s active participation: for instance, GPS data to research mobility (Krenn et al., 2011) or household meters to measure TV audiences (Mytton et al., 2016). Such passive data collection circumvents human memory limitations.

However, passively collected data cannot always replace survey data: the subjective information related to an event of interest (e.g., motivations), as well as part of the objective information (e.g., due to technological limitations) cannot be directly collected passively.

Sending a survey to a sample of participants in the moment an event of interest is detected using passively collected data has the potential to add the missing information that cannot be collected passively, reducing recall errors. These in-the-moment surveys decrease the time gap between the event and the data collection process compared to conventional surveys.

In this paper, we focus on in-the-moment surveys triggered by metered data. This type of data is obtained through a tracking software (a “meter”) willingly installed by a sample of participants on their browsing devices (PCs, tablets and/or smartphones) to record their online behaviors (e.g., visiting a particular website).

Metered data have been used, for instance, to predict voter turnout (Bach et al., 2021) or to study the effect of Facebook on the public agenda (Cardenal et al., 2018). Sending in-the-moment surveys triggered by metered data has the potential to combine the reliability of metered data in the online event detection with the flexibility of surveys to collect a wide variety of information, while reducing the impact of recall errors.

Metered data are currently collected by different opt-in online panels (e.g., Gapfish, Netquest, Respondi, Yougov) that ask some of their panelists to install a meter in at least one of their browsing devices. Such panels are known as “metered online panels” (Revilla et al., 2021). Even if sending in-the-moment surveys to metered panelists could allow investigating problems that, so far, could not be researched, previous literature shows that the willingness to participate in metered panels is usually quite low, from 3.6% to 42.1% depending on the specific panel, country, and device (Kissau and Fischer, 2016; Revilla et al., 2019; Revilla et al., 2021; Van Duivenvoorde and Dillon, 2015).

Asking panelists to participate in in-the-moment surveys in addition to installing the meter could reduce participation rates even more, which may compromise sample sizes and representativeness. This paper aims to shed light on the willingness to participate in this kind of in-the-moment survey, as well as on how such willingness is influenced by four factors (survey length, incentive level, invitation lifetime and triggering activity; see section 3) and by differences among individuals. To do so, a conjoint experiment was developed on a sample of metered panelists in Spain.

## Background

### Existing in-the-moment surveys

In-the-moment surveys have been widely used offline: for example, surveys of people leaving polling stations to predict an election outcome (Frankovic, 2012), or satisfaction surveys distributed in the train by railway companies. The online equivalent also exists: for instance, when connecting to a website to buy a train ticket, a pop-up window sometimes appears asking the user to participate in a survey related to the purchase. Furthermore, in-the-moment surveys have also been used in psychology (e.g., the Experience Sampling Method; see van Berkel et al., 2017) and audience research (e.g., coincidental surveys measured radio audiences in the 1930s, interviewing individuals who were contacted – by chance – in a moment when they were listening to the radio; see Lamas, 2005).

However, these in-the-moment surveys, both offline and online, correspond to very specific situations where the detection of individuals experiencing the event of interest and the feasibility of surveying them are particularly convenient. In fact, some of these applications (e.g., coincidental surveys) were abandoned due to the increasing difficulty of detecting individuals. Additionally, these examples are usually “one-shot” surveys, not allowing follow-up or control of the sample composition.

Focusing on in-the-moment web surveys triggered by passively collected data sent to members of an online panel should avoid the operational problems of finding participants and has the potential to extend the expected advantages of in-the-moment surveys to broader and more accessible samples. For instance, geolocation data have been used to trigger surveys based on participants’ physical location (Clemens and Ginnis, 2017; Haas et al., 2020; Wray et al., 2019). Similarly, smartphone sensor data have been used to trigger surveys based on participants’ physical activity (Lathia et al., 2013).

Metered data is another form of passively collected data that can trigger surveys to research participants’ online behaviors. However, the availability of samples is limited by the willingness to participate. Little research has been done about such in-the-moment surveys with one notable exception: Revilla and Ochoa (2018) used a pop-up to invite metered panelists in Spain to take part in a survey when a visit to a website of interest was detected through metered data. However, because only 18 persons completed the survey, valid conclusions could not be drawn.

Nevertheless, there is a lot of previous research about participation in conventional surveys, as well as in additional research tasks, which might help in understanding the willingness to participate in in-the-moment surveys.

### Willingness to participate in conventional surveys and additional research tasks

A number of theories have been suggested as explanations for survey participation (Albaum and Smith, 2012). One of the most prominent is social exchange theory (Blau, 1964) that, when applied to survey research (Dillman et al., 2009), suggests that people participate in surveys when they expect and trust that the perceived rewards outweigh the expected costs. Thus, there are three factors to be considered: costs, rewards, and trust.

For conventional web surveys, Keusch (2015) extensively covers the existing knowledge about factors influencing participation. Regarding additional non-survey research tasks (e.g., sharing sensor data from mobile devices), previous research has studied both willingness to participate and actual participation but under very different conditions (e.g., different technologies and duration of the tasks), making any comparison difficult. This previous research shows that there is a wide variation of willingness depending on the task (e.g., from 11.8% to 73.7% in Revilla et al., 2019). Different parameters have been found to affect such willingness: the efforts required to complete the task (Gatny et al., 2013), privacy concerns (Singer, 2011), incentives (Ochoa and Revilla, 2018), personal interest in the researched topic (Esser, 1986), sponsoring and survey provider reputation (Fang et al., 2012), as well as sociodemographic variables, attitudes, and personality traits (Pinter, 2015).

However, there is not a clear consensus on the size of the effect of these parameters. Moreover, whether these parameters also affect the willingness to participate in in-the-moment surveys still needs to be studied.

### Unique features of in-the-moment surveys compared to conventional surveys

According to social exchange theory, members of metered panels should have already assessed as positive the trade-off between rewards and costs of participating in conventional surveys and sharing metered data. Thus, we focus on the differences of costs and rewards between in-the-moment and conventional surveys, as well as the extent to which trust could be affected.

Regarding the additional costs, participants need to accept being notified about the survey by some sort of instant messaging system (e.g., SMS, app push notifications, or browser plug-in pop-up notifications), or, in cases using regular e-mails, activate the inbox e-mail notification in their smartphones (Ochoa and Revilla, 2022). Both the setup of these messaging systems and being interrupted when an in-the-moment survey is available can represent extra costs for the participants. However, in some cases, the survey invitation might not be perceived as an interruption, if it occurs at the end of an online activity and the participant has time available (for instance, right after buying a product online). Besides, given that participation must occur within a time limit, in-the-moment surveys diminish one of the main advantages of web surveys compared to interviewer-based surveys: the possibility for participants to choose their own timeline for completing the survey (Albaum and Smith, 2012).

Regarding rewards, as in-the-moment surveys are triggered by an action undertaken by the participants, better relevance and interest can be expected compared to conventional surveys. Research has shown that response rates are higher when the topics are of interest (Groves et al., 2004).

If the balance between additional costs and benefits of in-the-moment surveys is negative, the panel company has the option to increase the incentives for participating compared to conventional surveys. Such balance may change depending on different parameters of the surveys, such as its length or the available time to participate.

## Research questions, hypotheses, and contribution

The purpose of this study is to assess the willingness of metered panelists to participate in in-the-moment surveys triggered by online behaviors. More precisely, we propose the following research questions and hypotheses, taking into account that our target population are members of a metered panel who answer surveys regularly, are currently sharing metered data, and know the fieldwork company incentivization rules (e.g., surveys’ incentives are proportional to their length).

- *RQ1* – To what extent are metered panelists willing to participate in in-the-moment surveys triggered by metered data under different conditions?

In-the-moment surveys should not raise additional concerns besides those related to sharing metered data and taking regular surveys, except that in-the-moment surveys could make some panelists more conscious of the actual implications of sharing metered data. Revilla et al. (2021) found that 72.6% of those not willing to install a meter reported privacy concerns as the main reason, and another 7% raised the issue of trust. However, these additional concerns are expected to have a minor effect on most panelists’ willingness to participate. Thus, our hypothesis is that the willingness will be overall high (*H1*).

- *RQ2* – How do the attributes of in-the-moment surveys triggered by metered data influence willingness to participate?

We consider the following four attributes: survey length, incentive level compared to a conventional survey, invitation lifetime (maximum time allowed to start the survey after the invitation is sent), and triggering activity (the activity that, once detected, triggers an invitation to participate in a survey).

The first two have been selected because their influence on the willingness to participate is expected to change compared to a conventional survey, and the last two because they are specific to in-the-moment surveys. Other potential attributes, such as the invitation method used to notify participants about a survey, are not studied since it is quite easy in practice to use several methods at the same time.

Everything else being equal, a lower willingness is expected for longer surveys (*H2a*), lower incentive levels (*H2b*), shorter invitation lifetimes (*H2c*), and triggering activities raising more privacy concerns (*H2d*) and/or more sensitive to interruptions (*H2e*).

Larger effects on the willingness to participate are expected for survey length and incentive level than for invitation lifetime and triggering activity (*H2f*), which are expected to be more relevant for the actual participation than the willingness to participate, mainly due to practical issues (e.g., the invitation to take the survey might not be seen in time).

- *RQ3* – Are there significant differences in the willingness to participate among panelists with different profiles in terms of sociodemographic characteristics, attitudes, personality traits, and panel experience?

Among the sociodemographic characteristics, gender, age, and education are considered because previous research found that they influence the willingness to participate in some additional research tasks (Mulder and de Bruijne, 2019). However, in our case, and based on our target population, we only expect a significant (positive) effect of education (*H3a*) and not of gender (*H3b*) and age (*H3c*). Household size is added as a potential explanation not researched yet. Lower willingness to participate is expected for larger household sizes (*H3d*), due to more potential interruptions and less free time (e.g., taking care of children).

As for attitudes, previous research about online panelists’ willingness to perform additional research tasks suggests that frequency of sharing content in social media, trust in survey privacy, and trust in survey safety have an influence especially in tasks that require sharing sensitive information (Revilla et al., 2019). We therefore expect higher levels of willingness to participate for higher frequencies of sharing content in social media (*H3e*), trust in survey privacy (*H3f*), and trust in survey safety (*H3g*).

Regarding personality traits, the dimensions defined in the “Big 5” framework are studied. This framework has been used to find psychological characteristics related to the willingness to participate in research (e.g., Marcus and Schütz, 2005). Based on previous research about participation in panel surveys (Brüggen and Dholakia, 2010), we expect higher levels of willingness to participate for high agreeableness (*H3h*) and openness (*H3i*), while no significant influence is expected for consciousness (*H3j*), extraversion (*H3k*) and emotional stability (*H3l*).

Finally, panel experience was also considered a potential explanatory variable, as previous research found that past behavior influences nonresponse in online panel surveys (Haunberger, 2011). We hypothesize higher levels of willingness to participate for more experienced panelists (*H3m*).

- *RQ4* – What are the main reasons for deciding whether or not to participate stated by the panelists?

While the three first research questions are answered using a conjoint experiment (see section 5.1), in *RQ4* open questions are used to collect motivations and concerns that may not have been considered in the conjoint experiment. However, we expect the main reasons to correspond to the attributes considered in *RQ2* (*H4*).

By answering these research questions, we contribute to the existing knowledge in several ways. First, willingness to participate in in-the-moment web surveys triggered by metered data has not been researched yet. A better understanding of which attributes influence participation both contributes to the growing body of literature about willingness to participate in additional research tasks and helps design future in-the-moment surveys.

Second, we investigate attributes that may influence the willingness to participate in surveys that, to the best of our knowledge, have not been researched yet, such as the maximum time allowed to participate.

Finally, we use choice-based conjoint analysis (CBC, Louviere and Woodworth, 1983), which offers several advantages compared to conventional techniques and has been widely used in market research, but very little to investigate the willingness to participate in surveys or other research tasks. Keusch et al. (2019) used vignettes describing hypothetical passive mobile data collection studies, asking participants to rate their willingness to participate in each study, which is a form of conjoint analysis (not choice-based). Ochoa and Revilla (2018) used a CBC to research to what extent members of an online panel are willing to share different data types. However, the study did not consider in-the-moment surveys, and only varied the level of incentives and tasks.

## Data

The data were collected in Spain in May/June 2021 through the Netquest metered panel.^1^ The metered panel is a subset of the opt-in online survey panel, whose members voluntarily take part in surveys on a regular basis and are rewarded for it by means of points that can be redeemed for gifts. The longer the surveys, the larger the number of points. In order to be part of the metered panel, members of the opt-in online survey panel must install a meter in at least one of their browsing devices. In exchange, panelists earn two additional points per week for each device with the meter installed, up to a maximum of three devices.

The panelists invited to join the metered panel are not randomly selected from the survey panel. Instead, those panelists with higher likelihood to accept the meter installation according to an internal predictive algorithm are invited, leading to an installation rate of 29.3% in Spain (Revilla et al., 2021). Therefore, metered panelists are normally more experienced and engaged: the panelists analyzed in this study have been in the survey panel for 7.6 years on average and completed 378 surveys, which means approximately one participation per week. All in all, we focus on individuals with long experience in surveys, already sharing metered data and better incentivized than regular survey panelists.

The objective was to get a sample of 800 metered panelists. Quotas for gender, age, and education, were defined to reproduce the proportions of the online population in Spain according to the National Statistics Institute of Spain.^2^ From the 1900 panelists invited to the survey, 1701 started it (89.5%), 804 were considered valid participants, while 850 were discarded without taking the full questionnaire for different reasons: exceeding the quotas (777), not giving their explicit consent to participate (63), and not passing basic anti-fraud checks (10).

The average age of the 804 valid participants is 45 years, and 50% are women, 25% are mid-educated, and 35% are high educated; 68% have installed the meter on a desktop device, 86% on a smartphone, and 30% on a tablet. On average, participants have installed the meter in 1.9 devices. This means an additional incentive of around 15 points per month with respect to the average 45 points per month that panelists earn participating in conventional surveys.

## Methods

### A conjoint approach

In order to answer *RQ1*, *RQ2*, and *RQ3*, we asked metered panelists about their willingness to participate in in-the-moment surveys using a CBC analysis. Conjoint experiments (Green et al., 2001) estimate causal effects of multiple attributes on hypothetical choices or evaluations (Bansak et al., 2021). This method presents profiles, for example of products (Orme and Chrzan, 2017) or candidates (Carlson, 2015), with randomly assigned attributes and asks respondents to evaluate them. The random assignment of profile characteristics allows researchers to identify the influence of attributes on a participant’s decision.

CBC offers two key advantages for the purpose of this research. First, CBC is particularly suited to predict individuals’ decisions when the options offered include multiple desirable and undesirable attributes, forcing respondents to make trade-offs, as is normally the case in real life. Second, CBC measures the extent to which each attribute contributes to the choice, allowing researchers to predict the share of preference for each potential combination of attributes (Rao, 2014). However, these advantages come at the expense of an increased cognitive burden for respondents (Maddala et al., 2003), as CBC questionnaires require asking participants repeated choice tasks. Due to the trade-off between statistical efficiency and cognitive burden, it is recommended to use a maximum of six attributes (Lilien et al., 2013) with 2–5 levels per attribute (Lilien et al., 2013).

### Design of the conjoint experiment

First, a general description of what in-the-moment surveys are and the actual implications of participating in them was shown. Participants were specifically informed/reminded about the fact that they were already sharing metered data and that such data could be used to trigger in-the-moment surveys.

**Fig. 1:**
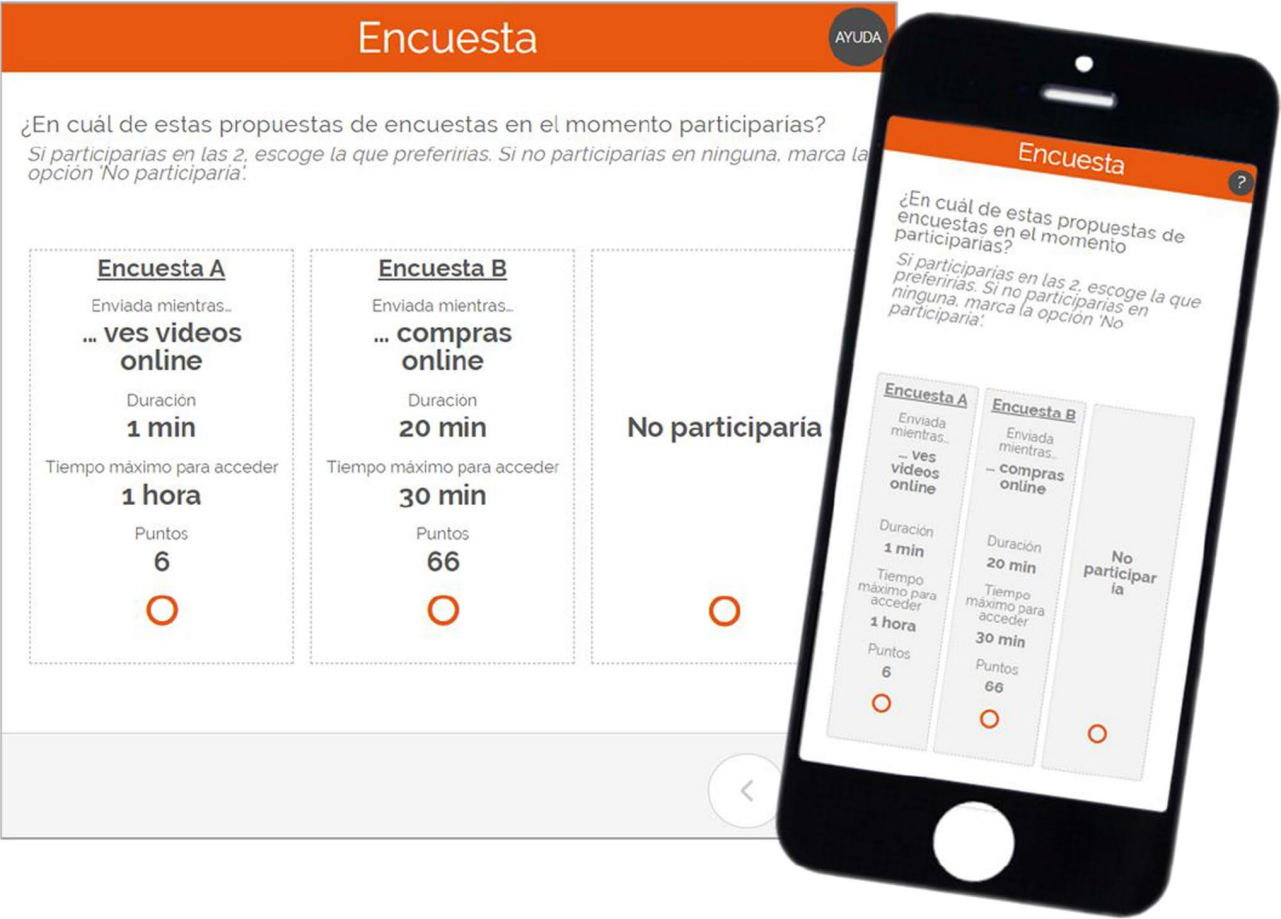
Example of a conjoint question

Then, the conjoint experiment proposed seven questions, each asking the respondents in which of the two different proposals of in-the-moment surveys they would participate. Each proposal included a combination of the four attributes considered in *RQ2*. Their order within the proposal was randomized for each participant. The option “I would not participate” was offered too (screenshots available in Fig. 1).

The number of questions, attributes, levels, and proposals were chosen to ensure the stability of estimations at an aggregated level (Orme, 1998), while limiting the cognitive burden typically caused by the repetitive nature of the conjoint questions. The different proposals shown in each question to each participant (i.e., combination of attributes levels) were designed to optimize D-efficiency (Kuhfeld, 2010) by means of a proprietary algorithm that follows the general method for constructing efficient choice designs (Zwerina et al., 2010).^3^

For each of the four attributes included in the conjoint experiment, we defined the following levels:

- Survey length: Previous research suggests that the maximum survey length that panel members are willing to participate in is around 20 min (Revilla and Ochoa, 2017), which is also considered the average adult attention span (Cape and Phillips, 2015). Additionally, in-the-moment surveys are expected to be short in general, as they must be answered within a time limit. Therefore, considering the recommendations about the maximum number of levels per attribute and that participants tend to round survey durations when asked about them (Revilla and Ochoa, 2017), five levels were tested: 1, 5, 10, 15, and 20 min.
- Incentive level compared to a conventional survey: Incentive levels were set using conventional surveys as a reference to allow participants to evaluate only those characteristics that are specific to in-the-moment surveys. The levels tested were: 1, 1.5, 2, 3 and 4 times the incentive for a conventional survey of the same length. Since the incentives increase response rates at a declining rate (Singer and Ye, 2013), we fixed the maximum at four times the incentive level of a conventional survey: little improvement is expected for higher levels, while the costs might become prohibitive.
  Incentive levels were not shown as such to participants. Instead, they saw the number of points that they would get for answering the survey. For instance, a conventional 10-min survey would be rewarded with 12 points according to the existing panel policy; if the incentive level was 1, the participants saw that they would get 12 points, whereas if the incentive level was 2, they saw that they would get (2 x 12=)24 points.
  Therefore, survey length and incentive level are not correlated by design. Instead, both attributes are correlated with the total number of points offered to participants. In fact, it was possible to be offered the same number of points for taking a short survey (e.g., 8 min) and a high incentive level (e.g., x2) than for a long survey (e.g., 18 min) and a low incentive level (e.g., x1). In both examples, the total number of points offered are 20. However, in the former case, the effort required to get such points is greater.
  This design does not allow for measuring the effect of the total number of points on the willingness to participate as it was not the purpose of this research. Only the effect of the incentive level (compared to a conventional survey) is measurable.
- Invitation lifetime: We used six levels: 15 and 30 min, and 1, 2, 6, and 12 h from the moment they received the invitation. We considered 15 min as the minimum time to react to an invitation. Moreover, we set a maximum of 12 h because (1) according to SurveyMonkey, 41% of the total responses to online surveys are gathered within 24 h;^4^ thus, in-the-moment surveys should propose shorter invitation lifetimes, and (2) human memory is cleaned of irrelevant information when people sleep (Izawa et al., 2019), so we wanted to avoid as much next-day participation as possible after the event of interest.
  Non-equidistant levels were set to measure shorter lifetimes with better precision, as people tend to forget information more rapidly right after the events (Tourangeau, 1999).
- Triggering online activities: There is no standard classification of online activities and, even if there were one, it would go beyond what can be measured using a conjoint analysis (Orme and Chrzan, 2017). Therefore, we decided to focus on five of the most popular online activities:
  - *Using social media*
  - *Reading online content*
  - *Watching online videos*
  - *Looking for information*
  - *Shopping online*
  These activities are expected to raise different levels of privacy concerns and sensitivity to interruptions, which in turn may lead to different levels of willingness to participate.
  As the evaluation of privacy and sensitivity to interruption for each activity may vary from one person to another, after the conjoint questions, participants were asked to evaluate each activity using seven-point rating scales. Such information was used in a regression analysis to explain differences in the willingness to participate depending on the triggering activity.
  Table 1 shows our expectations regarding (1) the levels of privacy concerns and sensitivity to interruptions for each activity and (2) the willingness to participate. Shopping online and watching online videos are expected to be the activities producing lower levels of willingness to participate due to potential privacy concerns in the former case (i.e., payment information in play) and high sensitivity to interruptions in both cases (i.e., interrupting a movie or an online purchase is expected to be more annoying than, for instance, interrupting a reading).

**Table 1:** Expected levels of privacy concerns, sensitivity to interruptions, and willingness to participate per triggering activity

Triggering activity	Privacy concerns	Sensitivity to interruptions	Willingness to participate
Shopping online	High	High	Low
Watching online videos	Low	High	Low
Using social media	Low	Low	High
Looking for information	Low	Low	High
Reading online content	Low	Low	High

### Full questionnaire

The final questionnaire included up to 57 questions that were asked in an online survey optimized for mobile devices. The average time to complete the questionnaire was 11.6 min and the median 9.7 min.

An English translation of the questionnaire and screenshots are available online in the supplementary material.^5^

Respondents could continue without answering the questions, except those used to control quotas and filter other questions. A warning message was shown to 92 participants who tried to skip a question when multiple questions were presented on the same page. Following the panel’s usual practice, going back was not allowed.

In this study, besides the seven conjoint questions, we used two open questions about the potential reasons to participate or not in in-the-moment surveys to answer *RQ4*. Moreover, we also used sociodemographic, attitudinal, and personality questions to identify differences in the willingness to participate among participants. Frequency of sharing content on social media, trust in survey privacy, and survey safety were measured as in Revilla et al. (2019). Five questions were selected from the Spanish adaptation (Martínez Mompó, 2015) of the “Big 5” Personality Trait Short Questionnaire in adults (Morizot, 2014), similar to those already used by Revilla and Höhne (2020) to research survey length preferences by panelists.

The number of past participations in surveys was used as a measure of the panelists’ experience. However, these data were not asked in the survey but directly provided by the panel company.

### Analysis

The analyses were performed using R 4.1.2. The scripts and the data are available online in the supplementary material.

#### Choice model

The participants’ answers to the conjoint questions were analyzed using a mixed logit model (McFadden and Train, 2000). Mixed logit assumes that individuals, when faced with a choice among competing alternatives, attempt to maximize their utility. The utility provided by each alternative is specified to be a linear combination of the utility of the attribute levels included in it, plus an unobserved utility (caused by unobserved factors) that is assumed to be independently, identically distributed type I extreme value. This choice behavior results in the multinomial logit formula to predict choice probabilities.

While the standard logit assumes fixed attribute level utilities for the entire population, mixed logit allows different utilities per individual. Individual utilities are specified to follow a set of upper-level probability distributions, whose means and standard deviations are estimated together with the individual utilities.

Individual attribute-level utilities, as well as the upper-level distribution parameters, were estimated using a Bayesian procedure developed by Allenby (1997) that requires estimating the entire probability distribution for each parameter by means of simulations. From the posterior distributions, 40,000 random draws were generated (using MCMC^6^) but only the last 20,000 were used for estimations, to ensure convergence to the actual distributions. Convergence was assessed by visually inspecting the stability of the estimations using a trace plot. To avoid autocorrelation among draws, only one every tenth draw was saved, which finally resulted in 2000 posterior draws (Train, 2003).

Following Orme and Chrzan (2017), point estimates, credibility intervals, and significant differences were assessed using the posterior draws, following a Bayesian approach. For instance, 95% credibility intervals were estimated, computing the 2.5th and 97.5th quantiles of the posterior draws. Similarly, to assess with 95% confidence (5% significance level) if utilities were significant, we counted how many of the posterior draws were greater than zero; if the proportion of positive draws was less than 0.05/2 or more than 1–0.05/2, we accepted the utility as significant (equivalent to a two-tail test). The same approach was used to assess significant differences between two utilities (or other quantities calculated on them, such as the attribute importance or the willingness to participate) but computing the proportion on the difference between the posterior draws of both utilities.

#### Utilities to assess influence on the willingness to participate

Utilities were first used to answer *RQ2*. As in any non-linear model, the interpretation of utilities is not straightforward, but offers a view on which attribute levels influence the willingness the most and in which direction.

Utilities are scaled to sum zero within each attribute and must be interpreted in relative terms. Positive utilities contribute to the willingness above the average, and vice versa. For instance, if we expect the attribute “incentive level” to have a positive effect on the willingness to participate, we should observe larger utilities for higher incentive levels. The attribute “none”, associated with the option “I would not participate” shown in each conjoint question, was coded differently. This attribute measures the utility of not participating, assuming implicitly a reference level of zero for participating. The utility of this attribute was used to assess the willingness to participate (see section 5.4.3).

Utilities were also used to evaluate the overall importance of each attribute on participants’ decisions. Relevant attributes are those that have more variation in utility among levels. The total variation within an attribute (i.e., largest utility minus smallest utility) over the sum of variations of all the attributes (excluding the “none” attribute) is a popular measure of attribute importance in choice models (Train, 2003).

#### Transforming utilities into willingness to participate

Once utilities are estimated, *RQ1* can be answered by transforming them into choice probabilities (that can be interpreted as the willingness to participate) using the multinomial logit formula. The inclusion of the option “I would not participate” allows for comparing the utility of accepting any potential in-the-moment survey proposal with the utility of not participating.

A different (more simplistic) approach to estimate the expected willingness to participate, based on the number of times that each participant selected the option “I would not participate”, was also used to confirm the results.^7^

#### Differences in utilities

To answer *RQ3*, differences in average utilities among groups of participants were used to find differences in attribute importance and willingness to participate.

The model specification used in this analysis forced utilities among individuals to follow an upper-level normal distribution, improving the estimation of individual utilities by overcoming the scarce information available per respondent in any CBC experiment.

However, this specification tends to hide differences among potential subpopulations, shrinking utilities towards a central value. According to Chapman and Feit (2015), adding covariates to the upper-level prevents the shrinkage, allowing the distribution mean to be a linear combination of different characteristics of participants, but at the expense of doubling the number of utilities to be estimated for each added binary covariate. To limit these problems, covariates were added to the model one by one, or in small groups of related covariates, and separate analysis were performed to find significant differences (at 5% significance level). The covariates analyzed together were: (1) age and education (negatively correlated in Spain, as older people are less educated), (2) survey attitudes (privacy and safety), and (3) the “Big 5” personality traits.

All the covariates were added to the model as categorical variables.^8^ To that end, numerical variables were grouped as follows: three groups for household size (small = 1, medium = 2–3, large ≥ 4), three groups for frequency of sharing social media (low = 1–2, medium = 3–5, high = 6–7), two groups for privacy and safety (low = 1–2 and high = 3–4 for privacy, while safety used a reversed scale), three groups for each “Big 5” personality trait (low = 1–2, middle = 3, high = 4–5) and three equal-sized groups for number of past participations in surveys (low = lowest 33% quantile, middle = middle 33% quantile, high = upper 33% quantile).

Each covariate produced 22 coefficients (one for each attribute level in the model) times the number of covariate levels minus one. In order to assess differences among groups of participants, the importance of attributes and the willingness to participate were calculated per group using the simulated posterior draws (see section 5.4.1.); significant differences among groups are also reported.

#### Open questions

Open questions were used to answer *RQ4*. The coding process looked for aspects of in-the-moment surveys that could lead panelists to participate or not, in order to detect whether there were other relevant aspects beyond those considered in the conjoint experiment.

Answers were coded by two native speakers. First, the main coder produced an initial codebook. After that, a secondary coder repeated the process using the same codebook. The intercoder reliability was 91% for the question on reasons for participation, and 97% for the one on reasons for non-participation. The reported results are those of the main coder, after review in light of those of the second coder.

## Results

### Factors influencing the willingness to participate

#### Preference among attribute levels

**Fig. 2:**
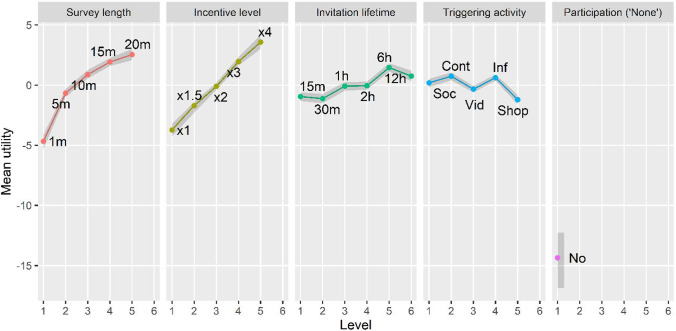
Mean utilities per attribute level and 95% credibility intervals. Note: Grey ribbons represent 95% credible intervals. The acronyms used for the triggering activities are the following: Soc = Using social media, Cont = Reading online content, Vid = Watching online videos, Inf = Looking for information, Shop = Shopping online

First, to answer *RQ2* we estimated the probability distributions of the attribute-level utilities. Figure 2 shows the mean utilities per attribute level (the shadowed band corresponds to a 95% credible interval), revealing how utility changes alongside attribute levels.

First, contrary to what we expected (*H2a*), the longer the survey, the larger the utility, but its increments are smaller as survey length increases. It is well established that short surveys are preferred to long ones (e.g., Keusch, 2015), but only if the same incentive level is offered. Therefore, considering the experimental design used, our result does not contradict the existing literature about the effect of the survey length on the willingness to participate. Since Netquest offers greater incentives for longer surveys, the positive effect of the incentive seems to outweigh the negative one of the survey length (at least up to 20 min), leading participants to prefer longer surveys (in order to get more points), even if those surveys must be completed in the moment.

Second, the higher the incentive level, the higher the utility and therefore, the willingness to participate. This is in line with our hypothesis (*H2b*).

Also in line with our expectations (*H2c*), larger invitation lifetime increases utility but with one inconsistency: utility for 6 h is significantly larger than for 12 h. From a rational point of view, we do not expect that having less time to participate is preferable. Therefore, either people prefer shorter times to force themselves to participate earlier (i.e., avoiding procrastination), or a significant part of the participants have misread the 12-h option. In any case, invitation lifetime is not the most relevant attribute for participants (see section 6.1.2).

As for the triggering activities, Table 2 shows them ordered from lowest to highest utilities (higher utility indicates higher willingness to participate; see section 5.4.3), together with the average scores of privacy concerns and sensitivity to interruptions reported by participants (0-6 scales; 6 indicating the highest concern). Participants who stated they did not do a particular activity were excluded from the analysis, as they represented only between 6 and 39 cases per activity.

**Table 2:** Utility of triggering activities and scores for privacy concerns and sensitivity to interruptions

Triggering activity	Mean utility	Privacy concerns	Sensitivity to interruptions
Shopping online	– 1.21	2.67	2.42
Watching online videos	– 0.33	2.50	2.13
Using social media	0.19	2.59	1.57
Looking for information	0.62	2.68	1.81
Reading online content	0.74	2.49	1.69

On the one hand, privacy scores show very limited variation (from 2.49 to 2.67) and, contrary to what we expected (see Table 1) “shopping online” does not seem to raise more privacy concerns than the other activities. Such limited variation could be due to self-selection: we are surveying highly experienced panelists that are already sharing metered data. It prevents observing any negative effect on the willingness to participate (no support for *H2d*).

On the other hand, sensitivity to interruptions show greater variation (from 1.69 to 2.42). As expected, “shopping online” and “watching online videos” are the two activities participants considered as most sensitive and the ones with lowest utilities, which indicates lower willingness to participate (support for *H2e*). Running a linear regression with fixed effects for participants (five observations per individual) results in a significant negative effect of sensitivity to interruptions (5% level).

Finally, regarding the attribute “none” (related to the option “I would not participate”), its strong negative utility (– 14.35) indicates that, on average, participants perceived not participating as very negative. In fact, only 3.7% of the answers to the conjoint questions were “I would not participate” and only 9.3% of the participants selected this option at least once.

#### Importance of attributes

In addition to which attribute levels are more or less preferred, we are interested in which attributes are more relevant for participants’ decisions. Table 3 presents the importance for each attribute.

**Table 3:** Importance of attributes

Attribute	Importance (%)	Percentile	
		2.5th	97.5th
Incentive level	38.2	35.5	40.6
Survey length	37.7	35.2	40.3
Invitation lifetime	13.7	11.0	16.2
Triggering activity	10.4	7.8	13.2

As we hypothesized (*H2f*), incentive level and survey length are the most influencing attributes (total = 75.9%). As survey length plays a role in the total amount of incentive that participants obtain when completing a survey, we conclude that panelists attach more importance to what they get in exchange for participating than to the particular drawbacks of in-the-moment surveys (i.e., limited time to participate, privacy issues and interruptions).

### Willingness to participate

Once attribute-level utilities have been estimated (section 6.1), such utilities can be used to make predictions about the willingness to participate in in-the-moment surveys (*RQ1*). However, a particular in-the-moment survey proposal must be specified.

To cover the whole range of willingness to participate we could expect, Table 4 shows the estimated willingness to participate for three different scenarios:

1. **Best scenario**. A proposal consisting of those attribute levels with highest average utilities: survey length = 20 min, incentive level = 4 times the equivalent conventional survey, invitation lifetime = 6 h, and triggering activity = reading online content.
2. **Worst scenario**. A proposal consisting of those attribute levels with lowest average utilities: survey length = 1 min, incentive level = the same as an equivalent conventional survey, invitation lifetime = 30 min, and triggering activity = online shopping.
3. **Average scenario.** A proposal consisting of the levels with median average utility within each attribute: survey length = 10 min, incentive level = 2 times the equivalent conventional survey, invitation lifetime = average utility of 1 and 2 h, and triggering activity = social media.

**Table 4:** Expected willingness to participate

Scenario	Mean willingness to participate (%)	Percentile	
		5th	95th
Best	94.7	93.7	95.8
Average	93.2	91.8	94.6
Worst	68.5	63.0	73.7

As expected (*H1*), there is high willingness to participate in all of the three scenarios, and even the worst scenario is accepted by 68.5% of participants. The difference between the average and best scenarios is minimal.

### Comparing panelists with different characteristics

Next, to answer *RQ3*, we compare the average attribute importance and willingness to participate for different groups of participants (see section 5.4.4) defined by the covariates of interest (see section 3). Table 5 shows such averages and indicates when differences among each pair of levels of each covariate are significant.^9^

**Table 5:** Importance of attributes and willingness to participate (both in %) per group of panelists

		Importance (%)				Willingness (%)		
		Survey length	Incentive level	Invitation lifetime	Triggering activity	Best	Avg.	Worst
**Sociodemographic**								
**Education**	Low	39.0*	38.7	14.2	8.2*	95.3*	94.4	69.2*
	Mid	31.9*	36.5	17.0	14.6*	91.5*†	91.9	54.6*†
	High	37.5	37.2	11.7	13.6	95.5†	93.6	77.3†
**Gender**	Male	36.1	35.9	15.6	12.4	94.6	93.2	65.9
	Female	37.7	40.0	13.3	8.9	94.6	93.7	72.6
**Age**	18-34	40.6*	38.2	11.0*	10.2	95.8*	94.3	65.3
	35-54	36.8	38.0	13.7	11.6	94.5	93.4	71.2
	55-74	32.8*	37.0	18.3*	11.8	92.9*	92.8	66.8
**Household size**	1	39.0	28.1*†	22.4*†	10.4	96.8*†	94.0	76.2
	2-3	37.2	39.1*	13.4*	10.2	94.3*	93.6	72.4
	>3	35.8	38.6†	12.7†	12.9	94.2†	92.7	63.2
**Attitudes**								
**Social media**	Low	37.4	39.2	13.7	9.7	94.9	94.3	66.3
	High	38.1	37.1	13.9	11.0	95.8	95.9	74.0
**Survey privacy**	Low	37.1	38.1	14.3	10.5	95.2	93.7	69.6
	High	37.2	36.7	12.6	13.5	93.0	90.9	62.9
**Survey safety**	Low	35.1	36.8	17.8*	10.3	95.9	94.6	71.6
	High	37.8	38.6	13.3*	10.3	94.7	93.2	68.3
**“Big 5” personality traits**								
**Agreeableness**	Negative	38.2	39.6	12.4	9.7	97.6*	96.8*	72.7
	Positive	37.7	38.0	14.3	10.1	93.1*	93.1*	68.4
**Openness**	Negative	35.2	38.4	12.2	14.2	94.9	90.5*	63.8
	Positive	37.2	38.0	14.7	10.1	94.7	94.3*	74.5
**Extraversion**	Negative	38.0	34.8	16.4	10.7	94.7	92.1*	59.3*
	Positive	37.8	38.6	14.4	9.2	94.3	94.8*	75.0*
**Stability**	Negative	42.0*	39.8	10.6	7.7	96.4*	94.6	68.7
	Positive	35.4*	38.6	14.0	12.1	94.3*	94.3	73.2
**Consciousness**	Negative	38.4	35.3	13.3	12.9	95.9	94.5	80.6
	Positive	37.2	38.6	14.1	10.1	94.3	93.3	69.1
**Experience doing surveys**								
**Participations**	Low	38.8	38.4	11.2*	11.7	94.0	95.1*	76.4
	Mid	39.0*	36.8	15.5	8.8	94.8	92.4*	72.9
	High	33.5*	38.4	16.2*	11.8	94.4	93.9	70.0

*,†: indicates a significant difference (5%) between two levels of a covariate.

#### Sociodemographic covariates

Education produces the largest number of significant differences. However, contrary to our expectations (*H3a*), such differences are between the participants with a medium education level and the other two groups. For instance, the willingness to participate is lower for the mid-educated group compared to the low and high educated ones, in all three scenarios (significantly in two of them). The size of the effect is also the largest among all the explored covariates: the willingness to participate for the mid-educated group is 54.6% in the worst scenario, the lowest level among all the different groups assessed. Potential explanations for this behavior include that mid-educated people may be educated enough to be aware of the potential risks of participating, but not enough to correctly assess the size of such risk (e.g., researchers are usually focusing on the aggregated survey results and not looking at a particular individual). However, further research is needed to find the actual explanation.

As expected, gender barely has an impact on the results (*H3b*).

Regarding age, it has a significant effect on the importance of two attributes: the younger group gives more importance to the survey length compared to the older one (40.6 vs. 32.8%) and less importance to the invitation lifetime (11.0 vs. 18.3%). That suggests that younger people should adapt better to the characteristics of actual in-the-moment surveys (shorter and faster than conventional surveys). However, as we hypothesized (*H3c*), these differences do not translate in relevant effects on the willingness to participate (one significant difference in the best scenario but inconsistent with the other two scenarios).

Regarding household size, in line with our expectations (*H3d*), a negative effect of larger groups on the willingness to participate is found for all three scenarios, even if it is significant only in the worst one (respectively 77, 71, and 62% for small, medium, and large households). The presence of more people within the household may relate to greater duties and distractions (such as caring activities, chores, face-to-face interactions), which reduce the time available to participate when answering from home.

#### Attitudinal covariates

The attitudinal covariates barely influence the results, contrary to our expectations (*H3e*, *H3f*, and *H3g*). Higher frequency in sharing content on social media and higher levels of trust in both survey privacy and safety are associated to higher levels of willingness to participate, but not significantly. However, the limited variations in these variables may have prevented any observation of the expected effect. Both variables were asked using four levels that were grouped into two groups (low and high trust), but only 6% of the participants were classified as “low trust in survey privacy” and 26% as “low trust in survey safety”. This might be because of the unique features of our target population (metered panelists).

#### “Big 5” personality traits

Although the personality traits have almost no influence on the importance panelists attach to each attribute (only one significant difference out of 20), there are several significant effects on the willingness to participate.

Agreeableness is associated with lower levels of willingness to participate in the three scenarios (significant in the best and average ones, 97.6 vs. 93.1%, and 96.8 vs. 93.1%, respectively).

Openness is related to a higher willingness to participate in two scenarios (significant in the average scenario, 90.5 vs. 94.3%).

Extraverted people present higher levels of willingness to participate in two scenarios (significant in the average and worst ones: 94.8% vs 92.1% and 75.0% vs 59.3%, respectively). Besides, it is the personality trait that presents the largest size effect (15.7 percentual points in the worst scenario).

Emotional stability produces a lower willingness in the best and average scenarios (significant in the former, 96.5% vs. 94.3%) but a higher willingness in the worst scenario.

Finally, consciousness does not produce significant differences in any scenario.

All in all, these results only support our hypothesis for consciousness (*H3j*). The positive effect hypothesized for openness (*H3i*) and the non-existent effect for extraversion (*H3k*) and emotional stability (*H3l*) are not supported by the data in all the scenarios. Additionally, the expected positive effect of agreeableness (*H3h*) not only finds no support, but evidence in the opposite direction. One reason could be that our hypotheses about personality traits were based on previous research on conventional surveys, not in-the-moment surveys.

#### Past experience as panelist

Contrary to our expectations (*H3m*), past experience as panelists produces a very limited effect. Other alternative variables^10^ to measure experience were explored, leading to similar results. However, the overall high level of experience in our sample could have prevented observation of this effect.

### Main reasons to participate or not stated by panelists

In order to answer *RQ4*, open questions asking about the reasons to participate or not in in-the-moment surveys aside from the four key attributes considered in the conjoint experiment were analyzed. Table 6 shows the percentage of respondents who mentioned each dimension. When respondents provided several reasons, we report all of them.

**Table 6:** Main reasons to participate or not in in-the-moment surveys

Main reasons stated to...			
...participate (*n* = 740)	%	... not participate (*n* = 63)	%
Incentive	58.8	Limited time	70.0
Like surveys	36.7	Privacy concerns	20.0
Have time	12.6	Interruption concerns	16.0
Appreciate short surveys	10.5	Do not develop the triggering activities	8.0
Other convenient aspects of in-the-moment surveys	12.3		

As expected (*H4*), answers to open questions do not reveal any relevant aspect in the participant’s decision not covered by the attributes used in the conjoint experiment and that could have been included in such experiment. When asked about reasons to participate, more than half of the respondents mentioned the incentive. Moreover, around one-third stated liking different aspects of surveys (e.g., sharing opinions, participating, knowing about new products). These are the answers we may expect to find in any opt-in online panel (Zhang et al., 2019) and are not particularly related to in-the-moment surveys. Additionally, 12.6% of participants mentioned having time available, for instance, due to unemployment or retirement. This answer may have been exacerbated by the COVID-19 crisis at the time the fieldwork was developed.

However, some respondents did comment on specific aspects related to in-the-moment surveys: 10.5% mentioned they appreciate surveys to be shorter and 12.3% indicated other convenient characteristics of in-the-moment surveys, like being related to activities they normally do, or being notified about their availability right in the moment (avoiding forgetting about them or procrastinating).

Regarding the reasons for not participating, lack of time and difficulties related to the limited time to participate were mentioned by most respondents. Privacy concerns, being interrupted and not developing the triggering activities were mentioned to a lesser extent.

## Discussion and conclusions

### Summary of main results

In this study, we investigated, among members of an opt-in metered online panel, the willingness to participate in in-the-moment surveys triggered by online events of interest using a conjoint experiment exploring four attributes of in-the-moment surveys (survey length, incentive level, invitation lifetime, and triggering activity) and open questions.

Regarding *RQ1*, we found that the willingness to participate is notably high: 93% in the average scenario and still 69% in the worst scenario considered. Thus, as expected, in-the-moment surveys seem to be well received by metered panelists.

Concerning *RQ2*, among the studied attributes, the incentive level and survey length are the attributes affecting most participants’ decision (76% of importance). Moreover, willingness to participate is higher for longer surveys (as they are better rewarded), higher incentive levels, longer invitation lifetimes, and for those triggering activities less sensitive to interruptions.

There are only few differences based on the participants’ characteristics (*RQ3*), most of them related to sociodemographic characteristics: in particular, people living in smaller households, with low or high education levels (versus medium levels). Among all the groups considered, the mid-educated panelists are those with the lowest willingness to participate (54.6%).

Finally, regarding *RQ4*, participants do not mention reasons for participating or not that are fundamentally different from the four attributes considered in RQ2, in addition to the usual reasons listed by previous research about participation in conventional surveys. The incentive seems to be a key driver.

All in all, the most problematic characteristics of in-the-moment surveys (limited time to participate, interrupting activities, privacy concerns) are well tolerated by most participants and do not inhibit high levels of willingness to participate in our sample of metered panelists.

### Limitations

First, we did not study actual participation. Experimental research is needed to assess potential differences between the willingness to participate and the actual participation. There are several reasons to observe differences between what people state and what they actually do (for a complete review, see Sun and Morwitz, 2010). In particular, respondents often produce a positive intention bias (Klein et al., 1997). We expect it to be particularly significant in in-the-moment surveys. The actual situation in which participants may find themselves when invited to participate is not known in advance. This is similar to any conventional survey. However, in-the-moment surveys require participation in a short time, making the situation in which participants are when receiving the invitation more determinant. Additionally, the average time needed to see the invitation to participate may play a crucial role but is not considered in this research.

Second, we focused on metered panelists, a very specific group of people. Thus, our results and, more specifically, the high levels of willingness to participate observed, should not be generalized to other target populations. We also used one specific opt-in online panel (Netquest) in one country (Spain). Other panels and countries may produce different results.

Third, participants were asked about their willingness to participate in one in the-moment-survey. Further research is needed to assess if the willingness would change in the case of being regularly asked to participate in such surveys.

Finally, the method used (CBC) is sensitive to the selection of the attributes. Although this limitation exists in almost any alternative method, CBC is particularly sensitive to the levels chosen to cover each attribute. Different selections may produce different evaluations of the attributes’ importance and, potentially, different levels of willingness to participate. This is particularly relevant for the attribute “triggering activity”. We used five categories of popular activities among dozens of potential activities. A different selection, aside from resulting in different utilities and levels of willingness to participate per triggering activity (like one would expect), could result in a different evaluation of the importance of the attribute.

### Practical implications

Our results can be used to guide researchers to design strategies and make practical decisions to develop in-the-moment surveys on samples from opt-in metered panels. Such results highlight the high levels of willingness to participate in samples drawn from metered online panels, even at similar levels of incentivization to those of conventional surveys. Survey length does not seem to be perceived as more critical than in conventional surveys; in fact, participants prefer longer surveys (at least up to 20 min, the maximum survey length studied) most probably in order to obtain more reward, despite the requirement to participate in the moment. The kind of online activity triggering surveys and the time allowed to participate seem to be of secondary importance. Therefore, researchers interested in maximizing participation rates for in-the-moment surveys should set the incentive level as high as possible and allow as much time as possible to participate while keeping it short enough to observe the expected advantages of asking people in the moment.

However, although incentives are highly appreciated by participants (attribute among those included in this research that received more attention), high incentive levels are not required to obtain notably high levels of willingness to participate. Nevertheless, it is very likely that actual participation may be substantially lower than the willingness to participate. For instance, participants might think that having a maximum time to participate is not a major problem, but then they may not manage to participate in time (do not see the invitation, cannot or do not want to interrupt the ongoing activity, etc.). In this case, incentives may be an effective lever to motivate panelists to overcome difficulties, used to reward specific actions to reduce such deviance (for instance, activating sound alerts related to mobile push notifications, or offering an extra-incentive for answering within the first 5 min to discourage procrastination).

Regarding sample composition, our research has not found large differences among participants in terms of willingness to participate. If actual participation does not deviate significantly from these results, in-the-moment surveys should not pose additional challenges in terms of representativeness to those already present in samples drawn from opt-in online panels in general and, more specifically, from metered panels.

All in all, this research suggests that in-the-moment surveys should be feasible if developed on samples drawn from metered panels, at least from the standpoint of potential participants’ willingness to participate. Practical information to design such surveys maximizing the expected participation have been provided. Real in-the-moment surveys are needed to prove the actual value of this method to improve data quality.

## Footnotes

1 www.netquest.com

2 www.ine.es

3 Further information on the experimental design is available at: https://osf.io/7vg2q/

4 https://www.surveymonkey.com/curiosity/time-to-respond/

5 https://osf.io/m4sw6

6 MCMC: Monte-Carlo Markov chain, a class of algorithms for sampling from a probability distribution. In Bayesian statistics, MCMC methods make it possible to compute large hierarchical models that require integrations over hundreds to thousands of unknown parameters.

7 Further information on the alternative analysis is available at: https://osf.io/f645q.

8 The analysis was also done using numerical covariates when possible, leading to similar results except for three differences in the willingness to participate that became non-significant for the “Big 5” covariates, and two differences that became significant for household size and log participations. For an overview of the full results, see the file “Descriptive-analysis.html” in the supplementary material: https://osf.io/8c4mz.

9 Full analyses are available at: https://osf.io/w6524.

10 We also considered the number of invitations to participate in surveys, number of valid surveys (i.e., participations excluding filtered surveys), years of membership, years sharing meter data and number of earned points. All these variables are highly correlated (correlation between 0.74 and 0.94). Limiting the time scope of these variables to the last 3 months produces similar results.
